# Development of genome-wide insertion/deletion markers and genetic diversity in *Sipunculus nudus* along the Beibu Gulf of China

**DOI:** 10.3389/fgene.2025.1542287

**Published:** 2025-04-10

**Authors:** Yuzhu Ni, Chunli Han, Yating Liu, Manman Li, Weijie He, Jialin Yang, Jie Zou, Huijing Peng, Pengliang Wang

**Affiliations:** ^1^ Guangxi Key Laboratory of Marine Environment Change and Disaster in Beibu Gulf, Beibu Gulf University, Qinzhou, Guangxi, China; ^2^ Guangxi Institute of Oceanology, Beihai, China

**Keywords:** *Sipunculus nudus*, genetic diversity, genome, insertion and deletion (InDel), molecular markers

## Abstract

*Sipunculus nudus*, a marine species of substantial medicinal and commercial importance, requires genetic enhancement to boost its production yield. However, progress in genetic research and selective breeding has been constrained by two critical limitations: the scarcity of available molecular markers and the absence of systematic genetic diversity assessments across China’s Beibu Gulf. To address these challenges, our genome-wide investigation identified 168,771 InDel variations, from which we developed 25,558 primer pairs. Experimental validation showed 82 out of 85 synthesized primers (96.47%) successfully amplified target regions, with 81 demonstrating polymorphism. Sixteen high polymorphic markers were subsequently employed to analyze 153 samples collected along the Beibu Gulf coastline, revealing 142 distinct alleles. The number of alleles, effective number of alleles, observed heterozygosity, expected heterozygosity, Shannon’s index and polymorphic information content ranged from 4 to 15 (mean of 8.875), 2.110 to 6.009 (mean of 4.110), 0.009 to 0.768 (mean of 0.232), 0.526 to 0.834 (mean of 0.734), 0.919 to 2.085 (mean of 1.576), and 0.440 to 0.816 (mean of 0.692), respectively. Population structure analysis revealed four genetically distinct subpopulations within the Beibu Gulf population. This delineation of population substructure provides critical insights for optimizing selective breeding programs and formulating germplasm conservation strategies in *S. nudus*.

## 1 Introduction

*Sipunculus nudus*, predominantly known as a sand worm (Edmonds, 1962), belongs to the family Sipunculidae within the phylum Sipuncula. They are widely distributed in coastal areas such as Guangxi, Guangdong, Fujian, and Hainan in China (Shao et al., 2015). In particular, the coastal areas of Guangxi have the richest genetic diversity of *Sipunculus nudus* (Liang, 1990; Maxmen et al., 2003); in general, they live in intertidal mudflat areas or sandy seabeds along the coast. They emerge during high tide and lurk in sand and mud caves during low tide and they feed on algae, organic matter, and plankton (Sun, 2020). Modern medical research has demonstrated that *S. nudus* have various properties including promoting wound healing (Zhang et al., 2011), enhancing cellular immunity (Cao, 2023), delaying aging (Shen et al., 2004), lowering blood pressure (Cai et al., 2023) and increasing antioxidant activity (Zhang and Dai, 2011; Li et al., 2016). *S. nudus* serves not only as a food source but also as a medicinal resource, highlighting its significant value and importance to us.

Escalating consumer demand for seafood, particularly *S. nudus*, driven by rising living standards and evolving nutritional trends, has triggered intensive harvesting operations. This unsustainable fishing pressure has resulted in significant erosion of genetic diversity, fundamentally undermining marine ecological equilibrium (Chen et al., 2020). Currently, conserving the genetic diversity of species is a key component of biodiversity conservation (Jiang and Ma, 2014). At the same time, genetic diversity is fundamental for genetic breeding (Zhang, 2013). Therefore, systematic investigations into *S. nudus* genetic diversity and evidence-based formulation of conservation strategies have become imperative for safeguarding these valuable natural resources.

Genetic diversity represents the spectrum of heritable variation existing among conspecific individuals within biological populations. This fundamental evolutionary property arises through four principal mechanisms: meiotic recombination of genetic material during inheritance, spontaneous mutations, interpopulation gene flow, and stochastic genetic drift (Brown, 1983).

Molecular markers are the useful tools to reveal genetic diversity. However, the molecular markers available in *S. nudus* included RAPDs (Song et al., 2011), SSRs (Wang et al., 2012) and fragments of mitodrial genome such as *COI* (the mitochondrial cytochrome coxidase subunit I) (Ning et al., 2012; Hsu et al., 2013), D-loop (Peng et al., 2017; Zhou et al., 2017), *cytb* (Song et al., 2017) and *16S* (Du et al., 2008). The types and number of molecular markers in *S.nudus* were so limited, although a genome sequence was reported (Zheng et al., 2023). The genetic diversity of *S. nudus* were seriously hindered.

Genetic diversity, the most crucial attribute of a population, serves as the foundation for adaptation to the environment and evolutionary improvement (Cui et al., 2012). Only few reports on the genetic diversity of *S. nudus* have been documented and focused on two areas. On one side, researchers revealed the genetic diversity of wild populations was higher than that of the cultured populations (Zhou et al., 2017). On the other side, several reports showed the higher level of genetic diversity of wild *S. nudus* along southern China including the provinces such as Fujian, Guangdong and Guangxi (Du et al., 2009; Song et al., 2011; Ning et al., 2012; Peng et al., 2017). However, some researchers reported the lower level of genetic diversity along the coasts of China (Song et al., 2017). All these conclusions were concerned with two major factors such as molecular markers and sampling.

In all reports above, the molecular markers only included RAPDs and some genes on the mitodrial genome. The RAPD markers lack stability because they have short primers (10 nt) and more binding sites (Lynch and Milligan, 1994). The conserved nature of animal mitochondrial genomes limits their utility in population-level genetic diversity assessments. Compounded by the scarce availability of SSR markers, these methodological constraints necessitate urgent development of novel molecular markers and comprehensive reevaluation of *S. nudus* genetic diversity to inform contemporary conservation strategies.

Insertions and deletions (InDels) are a common form of polymorphisms that correspond to the addition or removal of base pairs in the DNA sequence of an organism (Bhangale et al., 2005). In addition, InDels can also change gene expression by altering the phasing and spacing of DNA sequences in promoter regions (Cheung and Spielman, 2009). These InDel markers are often associated with important traits (Zhang et al., 2019). The molecular marker of InDels is valuable for molecular marker-aided breeding.

Our research consortium has successfully generated comprehensive genomic resources for *S. nudus*, including a fully assembled genome and multiple transcriptome profiles. This foundational sequencing work enables systematic development of high-resolution molecular markers, particularly Single-nucleotide polymorphisms (SNPs) and Insertion-Deletion (InDel) variants, for the future genetic analyses.

In this study, we aim to develop the InDel marker and assess genetic diversity and population structure in *S. nudus* across the Beibu Gulf.

## 2 Materials and methods

### 2.1 Identification of InDel markers

Eighteen transcriptomes of two tissues (guts and coelomic fluid), nine transcriptomes of each tissue, under ammonia nitrogen stress of 2954 mg/L for about 16 h and six transcriptomes of the two tissues in the seawater as control were sequenced and deposited in the Genome Warehouse at the National Genomics Data Center (accession number: GWHFDRQ00000000.1; https://ngdc.cncb.ac.cn/gwh). The quality of these sequences was evaluated via FastQC software. Low-quality reads, such as reads with ≥10% unidentified nucleotides (N), reads where more than 50% of the bases had quality scores lower than 20, and barcoded adapters, were removed. The high-quality reads were subsequently aligned to the genome via HISAT2. Variant calling was performed via the Unified Genotyper module of the Genome Analysis Toolkit (GATK) with default parameters. The InDels were extracted by the command line gatk SelectVariants--select-type-to-include INDEL.

For inDel validation and evaluation of genetic diversity, primers for InDels were designed via Primer3. A total of 85 primers (Supplementary Table S1) with identical or similar melting temperatures (Tm) between pairs were randomly chosen and synthesized by Sangon Biotech (Shanghai) Co., Ltd.

### 2.2 Distribution of InDels in chromosomes

To assess genome-wide InDel distribution patterns, we quantified InDels within consecutive 100 kb chromosomal intervals and calculated the density of InDel variants. To facilitate visual representation of genomic distribution, our analysis specifically focused on chromosomally anchored InDels while excluding those located on contigs.

### 2.3 Material collection and DNA extraction

**FIGURE 1:**
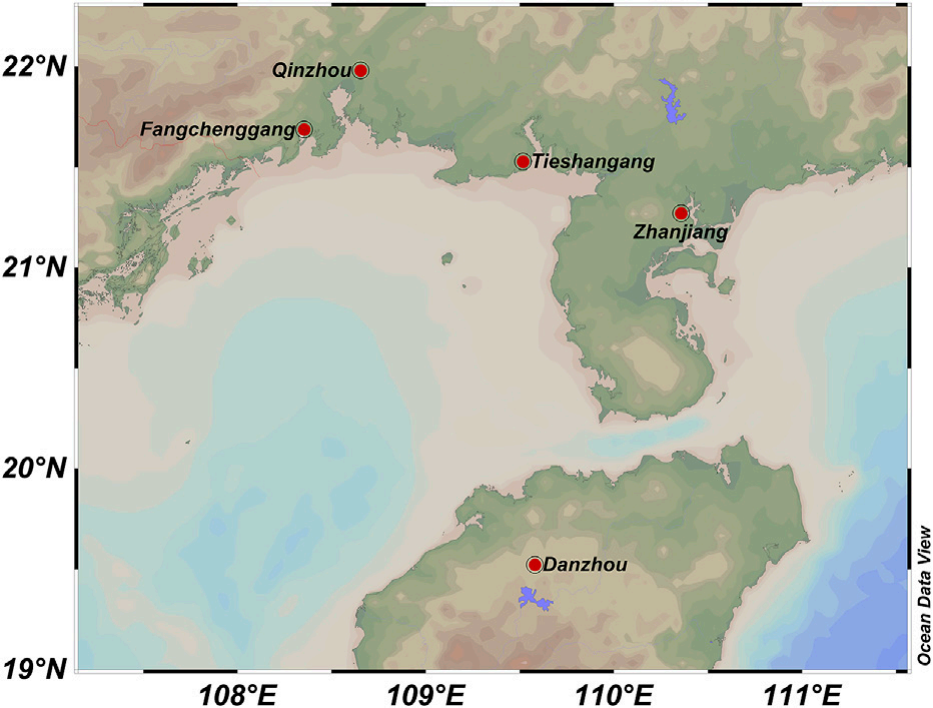
Sampling locations of *Sipunculus nudus* populations along the Beibu Gulf. Zhanjiang, Guangdong (ZJ, n = 50); Fangchenggang, Guangxi (FCG, n = 15); Danzhou, Hainan (HN, n = 32); Tieshangang, Beihai (TSG, n = 39); Qinzhou, Guangxi (QZ, n = 17).

A total of 153 wild individuals of *S. nudus* were collected from five geographically distinct populations along the Beibu Gulf including Zhanjiang (ZJ), Guangdong (n = 50; ZJ1 - ZJ50),Fangchenggang (FCG), Guangxi (n = 15; FCG1 - FCG15), Danzhou (HN), Hainan (n = 32; HN1 - HN32), Tieshangang (TSG), Beihai, Guangxi (n = 39; TSG1 - TSG39), Qinzhou (QZ), Guangxi (n = 17; QZ1 - QZ17) (Figure 1). All samples were transported to our laboratory. Genomic DNA was isolated using the TIANamp Marine Animals DNA Kit (DP324, Tiangen Biotech) following manufacturer protocols, diluted to 10 ng/μL working concentration, and stored at −20°C.

### 2.4 InDel-PCR analysis

The polymerase chain reaction (PCR) of the InDel markers was carried out at a volume of 10 µL. This reaction system contained 5 µL of 2 × TSINGKEMasterMix, 0.2 µL each of forward and reverse InDel primers (10 μmol/L), 1 µL DNA of 10 ng and 3.6 µL of ddH_2_O (Han et al., 2024).

The PCR program was as follows: 4 min at 95°C; 30 cycles of 45 s at 95°C, 45 s at Tm (depending on the primer) and 45 s at 72°C; a final extension of 7 min at 72°C and storage at 4°C. A 2 µL sample of the PCR product was separated on 8% nondenatured polyacrylamide gel. The gel consisted of acrylamide (39 acrylamide: 1 bisacrylamide) in 1×TBE buffer (90 mM Tris-boric acid, 2 mM EDTA; pH 8.0). The gels were 0.75 mm in thinness and had the dimensions of 16 cm × 36 cm. The electrophoresis conditions were 200 V for 2.5 h at room temperature. The gels were subjected to rapid silver staining for detection. The bands were recorded with their molecular weights by hand for subsequent analysis.

### 2.5 Genetic diversity and structure

PowerMarkerV3.2.5 (Liu and Muse, 2005) and GenAlEx6.5 software were used to analyze the parameters of genetic diversity, which included the number of alleles (*Na*), the effective number of alleles (*Ne*), the polymorphic information contents (*PIC*), the observed heterozygosity (*Ho*), the expected heterozygosity (*He*) and the Shannon index (*I*). Estimation of genetic differentiation (*Fst*) among populations was carried out via GenAlEx6.5.

We employed DARwin 6 to generate a phylogenetic tree file in the format of newick via neighbor-joining method with 1000 bootstrap replications. The clustering dendrogram was drawn using the iTOL web platform based on the newick tree file.

Structure 2.3.4 software was used to analyze the genetic structure of the population via an admixture model. The burn-in periods were set at 10,000 and 100,000 MCMC replicates. For each *K* value, the analysis was run 15 times. The number of subpopulations (*K*) ranging from 2 to 10 was assigned. The most likely K value was determined by the highest value of *ΔK,* obtained with STRUCTURE HARVESTER v0.6.94 (Earl and vonHoldt, 2012).

## 3 Results

### 3.1 Characteristics of genome-wide InDels

**FIGURE 2:**
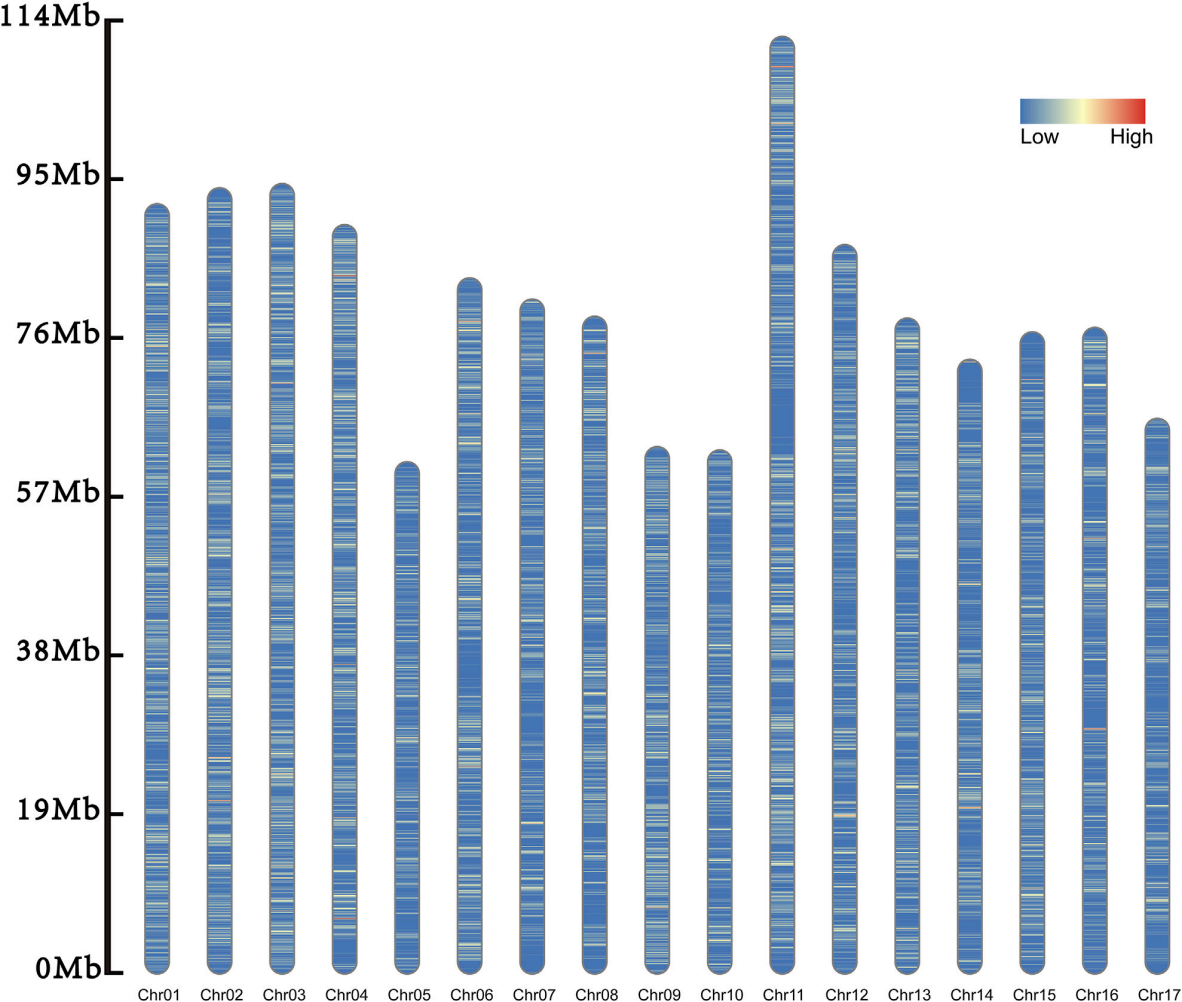
Distribution of inDel markers. The inDels on the 17 chromosomes are evenly distributed, and their densities are distributed from low to high and from blue to red.

In the genome, a total of 168,771 InDels were identified. Of them, 167,764 InDels were scattered on the 17 chromosomes (Table 1; Figure 2). The remained 1007 InDels were located on the 164 scaffolds.

**TABLE 1:** Distribution of InDels along 17 chromosomes according to size.

Chromosomes	InDels Size(bp)											
	≤10	≤20	≤30	≤40	≤50	≤60	≤70	≤80	>80	Total	Chr Len/Mb	InDel density
Chr01	12,752	573	187	54	34	22	4	0	10	13,636	96.581	141.187
Chr02	11,594	564	160	55	32	22	2	7	5	12,441	98.592	126.186
Chr03	12,791	608	193	68	30	17	12	0	13	13,732	99.119	138.540
Chr04	11,787	549	177	60	25	11	7	2	10	12,628	93.963	134.393
Chr05	6,246	209	66	20	12	6	3	1	11	6,574	64.235	102.344
Chr06	10,182	426	133	41	27	9	12	10	14	10,854	87.291	124.343
Chr07	7,804	364	100	44	19	8	5	3	4	8,351	84.624	98.684
Chr08	7,978	398	129	29	20	6	7	2	5	8,574	82.466	103.970
Chr09	8,331	349	115	33	20	9	1	3	8	8,869	66.127	134.120
Chr10	6,175	272	90	25	19	7	6	0	1	6,595	65.715	100.358
Chr11	13,685	677	215	66	41	10	8	7	15	14,724	117.579	125.226
Chr12	9,413	431	142	43	26	14	8	1	3	10,081	91.459	110.224
Chr13	8,856	415	151	36	22	13	3	1	4	9,501	82.238	115.531
Chr14	7,317	311	92	19	17	10	5	3	7	7,781	77.087	100.938
Chr15	8,448	357	101	38	29	8	3	2	5	8,991	80.506	111.681
Chr16	7,604	329	76	22	28	7	4	1	7	8,078	81.071	99.641
Chr17	6,004	230	71	15	13	4	5	1	11	6,354	69.669	91.203
Total	156,967	7,062	2,198	668	414	183	95	44	133	167,764	1438.323	1958.569

Among these chromosomes, Chr11 had the most InDels, with a total of 14,724 loci, while Chr17 had the smallest number, with only 6,354 loci. There was an average of 9,868.47 InDels every chromosome with the standard deviation of 2,604.44. The number of chromosomes with more than 11,000 InDels were five, while that harboring less than 7,000 InDels was three. This indicated that the number of InDels markers was unevenly distributed across the chromosomes.

Of all the chromosomes, Chr01 had the highest density of InDels (141.187/Mb) and Chr17 had the lowest density (91.203/Mb). The average density of InDels was 115.210/Mb with the standard deviation of 15.519. Therefore, we speculated the density of InDels was unevenly distributed too.

### 3.2 Screening of polymorphic InDels

A total of 25,558 primers were successfully designed (Supplementary Table S2). Five primers were selected every about one million InDels along each chromosome. In total, 85 primers were chosen for synthesis and then screened across six DNA templates. The results indicated that 3 primers did not amplify any bands; however, 82 primers were able to amplify bands, with 1 primer producing a single band. This finding indicated that the success rate of amplification was 96.47%, and the polymorphism rate was 95.29%.

### 3.3 Genetic diversity

Sixteen highly polymorphic primers were used to evaluate the genetic diversity of 153 samples of *S. nudus*. The results (Table 2) revealed that 142 alleles were detected. The number of alleles (*Na*) ranged from 4 to 15, with an average of 8.875. The effective number of alleles (*Ne*) varied from 2.110 to 6.009, with an average of 4.110. The observed heterozygosity (*Ho*) ranged from 0.009 to 0.768, with an average of 0.232. The expected heterozygosity (*He*) ranged from 0.603 to 0.834, with an average of 0.734. The Shannon index (*I*) ranged from 0.919 to 2.085, with an average of 1.576. The polymorphic information contents (*PIC*) ranged from 0.440 to 0.816, with an average of 0.692. The *Fst* value was 28% and the *Nm* value was 0.648, indicating larger among-population variance components. These results suggested that the genetic diversity of the population was relatively higher according to the report (Botstein et al., 1980) and the proportion of heterozygosity was significantly lower than the expected value in this study.

**TABLE 2:** The genetic diversity of *Sipunculus nudus* determined via InDel markers.

Loci	Left primer	Right primer	*Na*	*Ne*	*I*	*Ho*	*He*	*uHe*	*F*	*PIC*
Chr03-57264917	CAT​CCA​TGA​CGA​GGA​GGG​AAA​T	GCA​TTG​CAA​GAT​TCC​CAG​AGA​C	14.000	5.496	2.085	0.040	0.818	0.821	0.951	0.802
Chr04-71081678	ACC​TTC​AGA​GCT​CAC​CCA​TAA​T	AGT​GAA​CTG​AGC​ATT​GCC​TTA​T	14.000	6.009	2.076	0.159	0.834	0.837	0.809	0.816
Chr04-86496404	GGT​GCT​CAG​AAT​GCA​GGT​ATT​T	TGT​GTG​CAC​TGT​ATG​GCA​ATA​A	6.000	2.110	0.919	0.078	0.526	0.529	0.852	0.440
Chr04-88848678	CCA​CGC​TTT​GCA​TTA​CGT​TTA​A	CAT​AGG​GCC​ACG​GTA​ACA​TTA​C	9.000	4.790	1.745	0.520	0.791	0.794	0.343	0.762
Chr06-13466792	CAG​CAG​TGG​TTC​GAC​TCA​TTT​A	GCG​CCA​CCA​GTT​TCT​TGT​ATT​A	4.000	3.026	1.214	0.768	0.670	0.672	−0.147	0.608
Chr07-17389605	TGG​CCT​CTT​GAA​TGC​ACT​AAA​C	GGG​CTC​AAA​CAT​GCA​ACC​ATA​A	15.000	4.930	1.833	0.260	0.797	0.800	0.674	0.768
Chr07-32527104	GGG​ATG​AAC​AGG​TTG​GCT​TTA​A	ATC​CTG​TTG​CCA​CAC​ATG​ATA​A	5.000	2.699	1.114	0.291	0.630	0.632	0.538	0.556
Chr07-62670208	CCC​AAA​TTA​CAA​CCA​CCG​ACA​G	GAA​ACC​TGC​CAC​TGA​AGG​AAA​C	9.000	3.620	1.499	0.056	0.724	0.727	0.923	0.685
Chr08-9362134	TCG​GGT​AGA​CAT​CTT​TGA​CAC​T	GCA​GCT​CCA​AAC​AGT​GTT​ACA​T	11.000	4.356	1.784	0.304	0.770	0.773	0.606	0.743
Chr10-38115436	TTC​GTC​ACG​TGA​TTC​CCA​TAT​C	AAC​CAT​ACC​ACA​CCG​TCA​AAT​C	8.000	3.720	1.563	0.265	0.731	0.734	0.638	0.690
Chr10-2788932	GCA​CCT​CAA​CAT​GTA​CCA​TTC​A	AGA​GTG​GCC​TAA​ACT​TGG​ATT​C	12.000	5.069	1.913	0.009	0.803	0.806	0.989	0.778
Chr13-25794087	AAC​GAA​GGC​CTG​TCT​TAC​TTG​T	TCA​GCG​AGA​CGT​TCC​AGT​ATA​G	5.000	2.518	1.074	0.013	0.603	0.607	0.978	0.522
Chr13-42682127	CAA​AGC​TCG​ACA​CCC​TCA​AAT​C	ACA​CCC​TGT​TTC​CAC​CTG​TTT​A	7.000	3.799	1.458	0.116	0.737	0.741	0.842	0.691
Chr14-44111607	CAA​GCC​TCC​AGA​ACT​GTG​ATA​A	ATG​GTA​CCG​TGA​CTT​GCA​TTT​A	5.000	3.419	1.334	0.289	0.707	0.712	0.591	0.655
Chr16-5274098	GCA​TGT​ATC​ACG​CAG​AGC​TTA​G	GGT​TGC​GAT​TCA​CCC​TGT​TTA​T	10.000	5.318	1.909	0.204	0.812	0.816	0.749	0.788
Chr16-8297080	AAT​CAG​GCA​GCA​CAA​GGA​TAT​T	TCG​CAC​GAA​AGC​AGA​TGA​TAA​A	8.000	4.884	1.702	0.346	0.795	0.798	0.565	0.765
Average			8.875	4.110	1.576	0.232	0.734	0.737	0.681	0.692

### 3.4 Clustering

**FIGURE 3:**
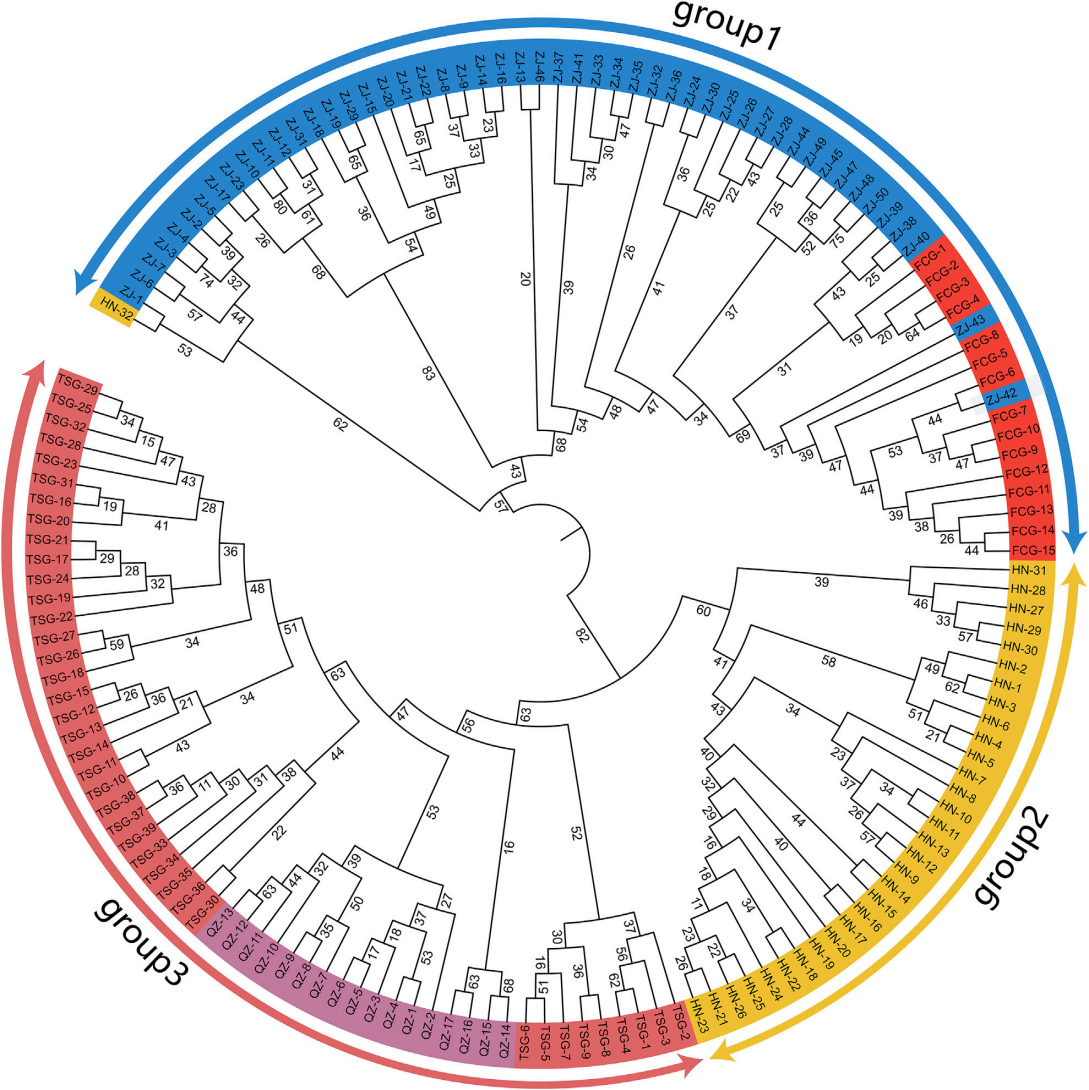
Clustering of the *Sipunculus nudus*. The blue represents the Zhanjiang population, the red represents the Fangchenggang population, the yellow represents the Hainan population, the dark red represents the Tieshan Port population, and the purple represents the Qinzhou population.

The results indicated that the samples from the five groups could be roughly divided into 3 distinct clusters (Figure 3), which agreed with the geographic distributions generally. The individuals from ZJ and FCG were clustered into group 1. All samples from FCG formed an independent branch besides five from ZJ in group 1; additionally, a sample from HN also clustered into group 1. The samples from HN were clustered into one branch (group 2). In the group 3, the individuals from TSG were split by those of QZ. We were surprised to find that the samples from FCG and ZJ were genetically closer to each other, while the samples from HN were closer to those from TSG and QZ. The results above suggested that the two provenances shared the closest genetic affinity between ZJ and FCG, whereas the genetic relationships among TSG, QZ and HN were relatively closer.

### 3.5 Genetic structure

**FIGURE 4:**
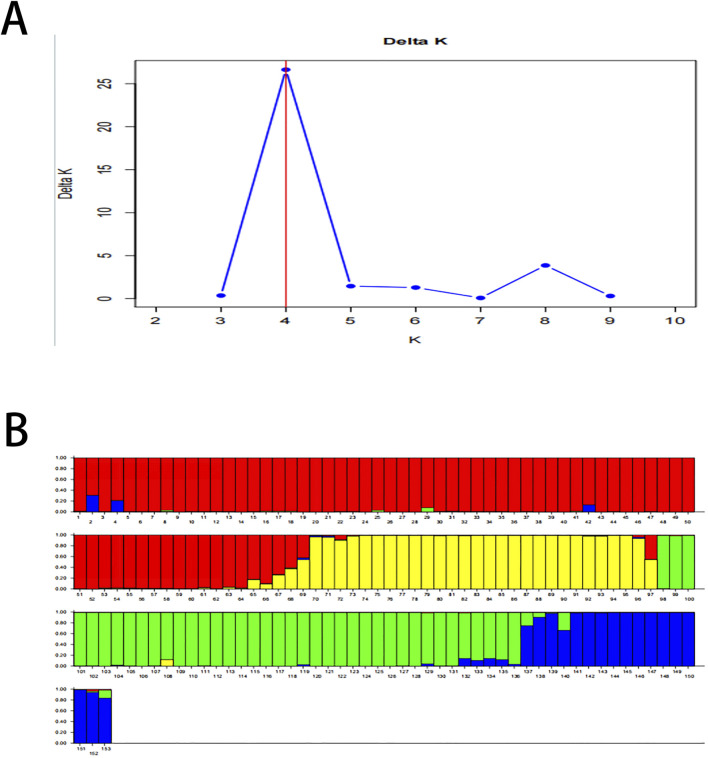
**(A)** Determination of the number of subpopulations (K). The K with the maximum value of delta K is 4, and the population is divided into 4 subpopulations. **(B)** Population structure of the genetic resources. Subpopulation 1 contains 68 samples (ZJ1-ZJ50, FCG1-FCG15, HN1-HN3, Red 1–68); Subpopulation 2 includes 29 individuals (HN4-HN32, Yellow 69–97); Subpopulation 3 consists of 39 individuals (TSG1-TSG39, Green 98–136); and Subpopulation 4 is composed of 17 individuals (QZ1-QZ17, Blue 137–153).

Structure 2.3.4 software was used to reveal the genetic structure in this study. The optimal number of subpopulations was determined using the DeltaK method. The results demonstrated that the *K* with the maximum *ΔK* at the turning point was 4 (Figure 4A). This suggested that 153 samples were divided into 4 subpopulations consisting of 68, 29, 39 and 17 samples in this study, respectively (Figure 4B). According to samples assigned, some samples from different provenances were placed into the same subpopulation. The subpopulation of ZJ mixed with 15 individuals from FCG and 3 individuals from HN.

## 4 Discussion

### 4.1 Genome-wide InDels

*S. nudus* is well known as a seafood for it’s higher nutritional value and delicious taste. However, *S. nudus* is in the wild state and the lower yield to meet with the demand in the market. The genetic improvement of it’s yield is essential. Molecular maker-aided breeding is an efficient and cost-effective way. Although various types of molecular markers, such as RAPDs (Wang et al., 2006) and SSRs (Wang et al., 2012), have been employed in the molecular research of *S. nudus*, the number of types of molecular markers is still relatively small compared with the number of molecular markers of oyster (Guo et al., 2023), posing challenges for the study of genetic resources. To date, high-quality molecular markers are quite lacking for *S. nudus*. InDels markers were useful tools, therefore identified and developed in this study.

InDel markers were distributed throughout the whole genome in this study, which were consistent with many species including *Micropterus salmoides L*. (Du et al., 2022) and grass carp (Nissa et al., 2021). However, the distribution of the InDels was uneven, with a higher density of InDels in some regions and a lower density in another. This might be due to the factor that the limited number of transcriptome samples used and their spatiotemporal gene expression resulted in only covering a part of the entire genome, which might strengthen the uneven.

### 4.2 Usability of InDel markers

According to previous reports (Zhang et al., 2019), InDel markers were associated with important traits, indicating that InDel markers were highly valuable. InDel markers have been reported in many model organisms, such as goats (Bi et al., 2022) and chickens (Brandström and Ellegren, 2007). In the present study, we developed a large mount of codominant InDel markers and selected 85 InDels for validation in *S. nudus*. Among the selected 85 InDels, 82 pairs could amplify products, but 81 pairs were polymorphic, indicating that this kind of marker was suitable for evaluation of genetic diversity and molecular marker-assisted breeding of *S. nudus*.

### 4.3 Genetic diversity

Sixteen polymorphic InDel markers were used to reveal the genetic diversity of *S. nudus*. The number and the effective number of alleles in the population were 8.875 and 4.110, which were significantly greater than the previously reported value of 3.750 and 2.106 revealed by SSRs (Wang et al., 2012). In addition, the Shannon’s index also surpassed the previously documents of 0.269 and 0.394 (Wang et al., 2006; Song et al., 2011). This might be attributed to increase in the number of genetic materials here in part and the useful tool of InDels developed in the present study in another part. At the same time, it was indirectly confirmed that the genetic diversity of *S.nudus* was the most abundant in Beibu Gulf.

In the present study, the *Ho* was significantly lower than *He*, which was not consistent with the report (Wang et al., 2012). The result showed that the homozygotes were left whereas those heterozygotes were removed. This might be the reason that the heterozygotes grew fast for their heterosis and be fished by human or went to death. Alternatively, this could be attributed to inbreeding consequences. In this case, the population composed of homozygotes has decreased adaptability to the environment, leading to a decline in the population’s survival ability.

### 4.4 Genetic structure

Differences in the genetic structure of a population reflect genetic diversity, which reflects the potential of a species to adapt to its changing environment (Niu et al., 2019). Genetic structure analysis based on the frequencies of alleles could reveal information on the origin and composition of lineages as well as the exchange of genetic materials (Liu et al., 2017).

The division of the entire population into 4 subpopulations revealed the existence of genetic structure within the population in this study. The inferred clusters corresponded closely to predefined populations.

The genetic structure is influenced by many factors including mating system, selection, gene flow and genetic drift et al (Wang et al., 2017; Hu et al., 2021).

The differences in mating systems have a significant impact on genetic structure. The *S. nudus* is a species that undergoes external fertilization. Both the sperm and eggs of *S. nudus* were released into the seawater, fluctuated, and mixed evenly; thus, the random fusion of sperm and egg to form a zygote is a manifestation of random mating. Consequently, we speculated that the mating system might not be the main factor of genetic structure.

In this study, the first subpopulation comprising 3 provenances indicated the presence of gene flow. The pattern was corroborated by the limited gene flow detected. Gene flow primarily occurs through two mechanisms: migration of genetic material or dispersal of gametes. Gametes are transported between populations via ocean currents, whereas genetic resources are introduced by human-mediated activities across geographical regions. However, the restricted gene flow ultimately resulted in distinct population structure, which was conformed by the pronounced variance components observed among provenances.

The high commercial pressure driven by the dual demand for *S. nudus* as a delicacy and medicinal resource has triggered unsustainable harvesting practices, exacerbating population declines. Overfishing could also result in alterations in gene frequencies. Hence, we speculated that human activities might be the main factor for the existence of genetic structure in the present study (Cheng et al., 2020).

The elevated observed homozygosity in this study could increase deleterious allele fixation risks, inducing inbreeding depression. Furthermore, restricted gene flow, especially in small or fragmented populations, amplifies genetic drift effects, causing stochastic allele frequency shifts. Such populations faced compounding threats: demographic instability, migration barriers, fluctuating sizes, and inbreeding escalation, collectively heightening extinction risks. Therefore, it is imperative to prioritize genetic resource assessment and protection for *S*. *nudus* to mitigate biodiversity risks.

## 5 Conclusion

In this study, a total of 168,771 InDels were identified in the genome, and 25,558 primers were designed. A subset was synthesized and validated. Approximately 96.47% of the primers worked successfully in *S. nudus*. Sixteen highly polymorphic InDels revealed abundant genetic diversity in the population and also revealed the genetic differentiation and gene flow that occurred among the provenances. Finally, the genetic structure was determined, and the population was divided into 4 subpopulations. The findings of the present study provided useful molecular markers and scientific evidence for the efficient utilization and effective conservation of the genetic resources of *S. nudus*.

## Data Availability

The datasets presented in this study can be found in online repositories. The names of the repository/repositories and accession number(s) can be found in the article/Supplementary Material.
